# Mushrooms as Possible Antioxidant and Antimicrobial Agents 

**Published:** 2012

**Authors:** Marijana Kosanić, Branislav Ranković, Marko Dašić

**Affiliations:** *Department of Biology, Faculty of Science, University of Kragujevac, 34000 Kragujevac, Radoja Domanovića 12, Serbia. *

**Keywords:** Mushrom exracts, Antioxidant activity, Antimicrobial activity

## Abstract

The aim of the study is to examine *in-vitro *antioxidant and antimicrobial activity of the acetonic and methanolic extracts of the mushrooms *Boletus aestivalis, Boletus edulis *and *Leccinum carpini*. Antioxidant activity was evaluated by using free radical scavenging activity and reducing power. In addition, total content of phenol and flavonoid in extracts were determined as pyrocatechol equivalent, and as rutin equivalent, respectively. As a result of the study acetonic extracts from *Boletus edulis *was more powerful antioxidant activity with IC50 value of 4.72 μg/mL which was similar or greater than the standard antioxidants, ascorbic acid (IC50 = 4.22 μg/mL), BHA (IC50 = 6.42 μg/mL) and *α*-tocopherol (IC50 = 62.43 μg/mL). Moreover, the tested extracts had effective reducing power. A significant relationship between total phenolic and flavonoid contents and their antioxidative activities was significantly observed. The antimicrobial activity of each extract was estimated by determination of the minimum inhibitory concentration by using microdilution plate method against five species of bacteria and five species of fungi. Generally, the tested mushroom extracts had relatively strong antimicrobial activity against the tested microorganisms. The minimum inhibitory concentration for both extracts related to the tested bacteria and fungi were 1.25 - 10 mg/ mL. The present study shows that tested mushroom species demonstrated a strong antioxidant and antimicrobial activity. It suggests that mushroom may be used as good sources of natural antioxidants and for pharmaceutical purposes in treating of various deseases.

## Introduction

Reactive oxygen species (ROS) are an entire class of highly reactive molecules derived from the metabolism of oxygen. At normal physiological concentrations ROS are required for cellular activities, however, at higher concentrations, ROS can cause extensive damage to cells and tissues, during infections and various degenerative disorders, such as cardiovascular disease, aging, and neurodegenerative diseases like Alzheimer’s disease, mutations and cancer (1, 2). 

Antioxidants, both synthetic or natural, can be effective to help the human body in reducing oxidative damage by ROS (3). However, at the present time, suspected that synthetic antioxidants such as butylated hydroxyanisole (BHA), butylated hydroxytoluene (BHT), tert-butylhydroquinone (TBHQ) and propyl gallate (PG) have toxic and carcinogenic effects (4). Therefore, the development and utilization of more effective antioxidants of natural origins are desired. In recent years, the antioxidant properties of numerous plants, lichens and mushrooms have been widely reported. In order to find new natural sources of antioxidants, our attention was focused on mushrooms. 

Mushrooms possess high contents of qualitative protein, crude fibre, minerals and vitamins. Apart from their nutritional potentials, mushrooms are also sources of physiologically beneficial bioactive substances that promote good health. They produce a wide range of secondary metabolites with high therapeutic value. Health promoting properties, *e.g. *antioxidant, antimicrobial, anticancer, cholesterol lowering and immunostimulatory effects, have been reported for some species of mushrooms. Both fruiting bodies and the mycelium contain compounds with wide ranging antioxidant and antimicrobial activities (5-8). Thus, the aim of this study is to examine *in-vitro *antioxidant and antimicrobial activity of the acetonic and methanolic extract of the mushrooms *Boletus aestivalis, Boletus edulis *and *Leccinum carpini*.

## Experimental

*Fungal materials*

Fungal samples of *Boletus aestivalis *(Paul.) Fr., *Boletus edulis *Bull. Fr., and *Leccinum carpini *(Schulzer) Moser ex Reid were collected from Kopaonik, Serbia, in June of 2010. The demonstration samples are preserved in facilities of the Department of Biology and Ecology of Kragujevac, Faculty of Science. Determination of mushrooms was done using standard keys (9-11). 

*Extraction*

Fresh fungal material was milled by an electrical mill. Finely ground mushrooms (50 g) were extracted using acetone and methanol for 24 h. The extracts were filtered and then concentrated under reduced pressure in a rotary evaporator. The dry extracts were stored at -18°C until used in the tests. The extracts were dissolved in 5% dimethyl sulphoxide (DMSO).

*Antioxidant activity*

*Scavenging DPPH radicals*

The free radical scavenging activity of mushrooms extracts was measured by using 1,1-diphenyl-picryl-hydrazil (DPPH). The method used was almost the same as the one used by other authors (12, 13), but was modified in details. Two mL of methanol solution of DPPH radical in the concentration of 0.05 mg/mL and 1 mL of extract were placed in cuvettes. The mixture was shaken vigorosly and left to stay at room temperature for 30 min. After that the absorbance was measured at 517 nm in spectrophotometer (Bibby Scientific Limited, Stone, UK.) Ascorbic acid, butylated hydroxyanisole (BHA) and *α*-tocopherol were used as positive control. Inhibition of free radical DPPH as percentage [I (%)] was calculated as follows:

I (%) = (A0 - A1 / A0) × 100

where A0 is the absorbance of the negative control and A1 is the absorbance of reaction mixture or standards.

The inhibition concentration at 50 % inhibition (IC50) was the parameter used to compare the radical scavenging activity. A lower IC50 means better radical scavenging activity.

*Reducing power*

The reducing power of extracts was determined by the method of Oyaizu (14). One mL of extracts were mixed with 2.5 mL of phosphate buffer (2.5 mL, 0.2 M, pH 6.6) and potassium ferricyanide [K3Fe(CN)6] (2.5 mL, 1%). The mixtures were incubated at 50oC for 20 min. After that, trichloroacetic acid (10%, 2.5 mL) was added to the mixture and centrifuged. Finally, the upper layer were mixed with distilled water (2.5 mL) and 0.5 mL of 0.1% ferric chloride (FeCl3). The absorbance of the solution was measured at 700 nm in spectrophotometer. Higher absorbance of the reaction mixture indicated that the reducing power is increased. Ascorbic acid, BHA and *α*-tocopherol were used as positive control.

*Determination of total phenolic compounds*

Total soluble phenolic compounds in the mushrooms extracts were determined with Folin-Ciocalteu reagent according to the method of Slinkard and Slingleton (15), using pyrocatechol as a standard phenolic compound. Briefly, 1 mL of the extract (1 mg/mL) in a volumetric flasc diluted with distilled water (46 mL). One milliliter of Folin-Ciocalteu reagent was added and the content of the flask was mixed thoroughly. After 3 min 3 mL of 2% sodium carbonate (Na2CO3) was added and then was lleft to stay for 2h with intermittent shaking. The absorbance was measured at 760 nm in spectrophotometer. The total concentration of phenolic compounds in the extract was determined as microgram of pyrocatechol equivalent by using an equation per milligram of dry extract, using the equation which derived from standard pyrocatechol graph as follows:

Absorbance = 0.0144 x total flavonoid (μg rutin equivalent/mg of extract) + 0.0556

*Total flavonoid content*

The total flavonoid content was determined by using the Dowd method (16). Two mL of 2 % aluminium trichloride (AlCl3) in methanol was mixed with the same volume of the extract solution (1 mg/mL). The mixture was incubated at room temperature for 10 min, and the absorbance was measured at 415 nm in spectrophotometer against blank samples. The total flavonoid content was detemined as microgram of rutin equivalent by using an equation that was obtained from standard rutin graph as follows:

Absorbance = 0.0144 x total flavonoid (μg rutin) + 0.0556

(R2 = 0.9992)

*Antimicrobial activity*

*Microorganisms and media*

The following bacteria were used as test organisms in this study: *Staphilococcus aureus *(ATCC 25923), *Escherichia coli *(ATCC 25922), *Klebsiella pneumoniae *(ATCC 70063), *Pseudomonas aeruginosa *(ATCC 27853) and *Enterococcus faecalis *(ATCC 29212). All the bacteria used were from the American Type Culture Collection (ATCC). Their identification was confirmed at the Microbiological Laboratory of Kragujevac, University of Kragujevac, Department of Biology. The fungi used as test organisms were: *Aspergillus flavus *(ATCC 9170), *Aspergillus fumigatus *(DBFS 310), *Candida albicans *(IPH 1316), *Paecilomyces variotii *(ATCC 22319), *Penicillium purpurescens *(DBFS 418). They were from the American Type Culture Collection and the mycological collection maintained by the Mycological Laboratory within the Department of Biology of Kragujevac, University of Kragujevac, Faculty of Science (DBFS). Bacterial cultures were maintained on Müller-Hinton agar substrates (Torlak, Belgrade). Fungal cultures were maintained on potato dextrose (PD) agar and Sabourad dextrose (SD) agar (Torlak, Belgrade). All cultures were stored at 4°C and subcultured every 15 days. 

The sensitivity of microorganisms to acetone and methanol extracts of the examined species of mushrooms was tested by determining the minimal inhibitory concentration (MIC).

Bacterial inoculi were obtained from bacterial cultures incubated for 24 h at 37°C on Müller-Hinton agar substrate and brought up by dilution according to the 0.5 McFarland standard to approximately 108 CFU/mL. Suspensions of fungal spores were prepared from fresh mature (3- to 7-day-old) cultures that grew at 30°C on a PD agar substrate. Spores were rinsed with sterile distilled water, used to determine turbidity spectrophotometriacally at 530 nm, and then further diluted to approximately 106 CFU/mL according to the procedure recommended by NCCLS (17).

*Minimum inhibitory concentration*

The MIC was determined by the broth microdilution method using 96-well micro-titer plates (18). A series of dilutions with concentrations ranging from 40 to 0.156 mg/mL for extracts were used in experiment against every microorganism tested. The starting solutions of extracts were obtained by measuring off a certain quantity of extract and dissolving it in DMSO. Two-fold dilutions of extracts were prepared in Müller-Hinton broth for bacterial cultures and SD broth for fungal cultures. The MIC was determined by establishing visible growth of microorganisms. The boundary dilution without any visible growth was defined as the MIC for the tested microorganism at the given concentration. As a positive control of growth inhibition, streptomycin was used in case of bacteria, ketoconazole in case of fungi. 

A DMSO solution was used as a negative control. All experiments were performed in triplicate.

*Statistical analyses*

Statistical analyses were performed with the EXCEL (version 11) and SPSS (version 13) software packages. To determine the statistical significance of antioxidant activity, student’s t-test were used. Pearson’s bivariate correlation test was carried out to calculate correlation coefficients (r) between the content of total phenolic and flavonoid and the DPPH radical scavenging activity. All values are expressed as mean + SD of three parallel measurements.

## Results and Discussion

*Antioxidant activity*

DPPH radical scavenging, reducing power, determination of total phenolic compounds and determination of total flavonoid content of the acetone and methanol extracts of the mushrooms *Boletus aestivalis, Boletus edulis *and *Leccinum carpini *were examined in this study.

The scavenging DPPH radicals of the studied extracts are shown in Table 1. 

**Table 1:** IC50 values of acetone and methanol extracts of *Boletus aestivalis, Boletus edulis *and *Leccinum carpini *by free radical scavenging method

**Samples**	**IC**50 **(μg/mL)**					
	**B. aestivalis**	**B. edulis**	**L. carpini**	**Ascor. acid**	**BHA**	***α*** **-tocoph.**
Acetone extracts Methanol extracts	8.63 187.73	4.72 212.47	67.89 202.47	4.22	6.42	62.43

The inhibition concentration at 50 % inhibition (IC50) was the parameter used to compare the radical scavenging activity. A lower IC50 meant better radical scavenging activity. Acetone and methanol extracts of the tested mushrooms showed a good scavenging activity on DPPH radical. There was statistically significant difference between extracts and control (P < 0.05). The IC50 values of all extracts ranged from 4.72 – 212.47μg/ml. Acetone extract from *Boletus edulis *showed largest DPPH radical scavenging activities (IC50 = 4.72 μg/ml) than those from other samples and greater than BHA and α-tocopherol. The scavengig activity was also good for the acetone extracts from *Boletus aestivalis *(IC50 = 8.63 μg/ml) and *Leccinum carpini *(IC50 = 67.89 μg/mL). Methanol extracts from tested mushrooms showed weaker DPPH radical scavenging activities than acetone. IC50 for the methanol extracts were 187.73 μg/mL for *Boletus aestivalis, *212.47 μg/mL for *Boletus edulis *and 202.471 μg/mL for *Leccinum carpini*.

The results of the reducing power assay of tested extracts are summarized in Table 2. High absorbance indicates high reducing power. Measured values of absorbance varied from 0.0014 to 0.0280. The reducing power of extracts increased contcentration dependently. Among the tested extracts, acetone and methanol extracts of *Boletus edulis *showed highest reducing power, followed by acetone extracts from *Leccinum carpini*. Other extracts showed weaker reducing power.

**Table 2:** Reducing power of acetone and methanol extracts of *Boletus aestivalis, Boletus edulis *and *Leccinum carpini*

**Samples**	**Absorbance (700 nm)**			
	**Extracts**	**1000 μg/mL**	**500 μg/mL**	**250 μg/mL**
*B. aestivalis*	Acetone Methanol	0.025 0.028	0.005 0.006	0.005 0.003
*B. edulis*	Acetone Methanol	0.008 0.009	0.005 0.007	0.002 0.003
*L. carpini*	Acetone Methanol	0.023 0.008	0.006 0.004	0.003 0.001
Ascorbic acid		2.226	0.957	0.478
BHA		3.465	1.681	1.651
*α*-tocopherol		2.887	1.651	0.808

Total phenolic and flavonoid constituents of tested extracts are presented in Table 3.

**Table 3:** Total phenolics and flavonoid content of acetone and methanol extracts of *Boletus aestivalis, Boletus edulis *and *Leccinum carpini*

**Samples**	**Extracts**	**Phenolics content** **(μg of pyrocatechol equivalent/mg of extract)**	**Flavonoid content** **(μg of rutin equivalent/mg of extract)**
*B. aestivalis*	Acetone Methanol	6.73 ± 1.065 4.91 ± 1.208	3.20 ± 1.099 1.53 ± 1.105
*B. edulis*	Acetone Methanol	8.14 ± 1.211 4.64 ± 1.318	4.93 ± 1.195 1.46 ± 1.128
*L. carpini*	Acetone Methanol	5.93 ± 1.341 4.69 ± 1.208	1.86 ± 1.213 1.48 ± 1.106

The amount of total phenolic compounds was determined as the pyrocatechol equivalent using an equation obtained from a standard pyrocatechol graph. Results of the study showed that the phenolic compound of the tested extracts varied from 4.64 to 8.14 μg of pyrocatechol equivalent. Highest phenolic compounds was identified in acetone extract of *Boletus edulis *at a 8.14 μg of pyrocatechol equivalent, followed by acetone extract of *Boletus aestivalis *with 6.73 μg of pyrocatechol equivalent.

The amount of total flavonoid compounds was determined as the rutin equivalent using an equation obtained from a standard rutin graph. As shown in Table 3, good flavonoid content was found in the acetone extract of *Boletus edulis *(4.93 μg of rutin equivalent) and acetone extract of *Boletus aestivalis *(3.20 μg of rutin equivalent). Other lichen extracts showed lower flavonoid content.

The tested mushroom extract and their IC50 values was correlated with total phenolic and flavonoid content (Figure 1). Notably negative correlation was established between the phenols and IC50 values of antioxidant activities (r = -0.93). Also, there is a good negative correlation between flavonoid compounds of the tested extracts and IC50 values of antioxidant activities (r = -0.83). These negative linear correlations prove that the sample with highest antioxidant contents show higher antioxidant activity with lowest IC50 values.

*Antimicrobial activity*

The antimicrobal activity of the tested mushrooms extracts against the tested microorganisms was shown in Table 4.

**Table 4:** Minimum inhibitory concentration (MIC ) of acetone and methanol extracts of *Boletus aestivaliss, Boletus edulis *and *Leccinum carpini*

**Samples**	**B. aestivalis**		**B. edulis**		**L. carpini **		**S - K**	
**Test organisms **	**A **	**M**	**A**	**M**	**A**	**M**		
*E. foecalis* *E. coli* *K. pneumoniae* *P. aeruginosa* *S. aureus* *A. flavus* *A. fumigatus* *C. albicans* *P. purpurescens* *P. verrucosum*	2.5 5 2.5 5 5 5 5 2.5 10 10	5 10 5 5 5 10 10 5 10 10	5 5 2.5 2.5 2.5 10 10 2.5 10 10	5 10 5 5 5 10 10 5 5 5	1.25 5 2.5 2.5 2.5 5 5 5 10 5	10 5 2.5 2.5 5 10 10 5 10 5	15.62 31.25 1.95 15.62 31.25 - - - - -	- - - - - 3.9 3.9 1.95 3.9 3.9

^a ^Minimum inhibitory concentration ( MIC ); values given as mg/mL for extracts and as μg/mL for antibiotics. Values are the mean of three replicate.

Antibiotics: K – ketoconazole, S – streptomycin.

The acetone and methanol extracts of the tested mushrooms showed relatively strong antimicrobial activity. The MIC for both extracts related to the tested bacteria and fungi were 1.25 - 10 mg/mL. Generally, the acetone extracts exerted stronger antimicrobial activity than methanol extracts.

The maximum antimicrobial activity was found in the acetone extract of the mushrooms *Leccinum carpini *against *Enterococcus foecalis *(MIC = 1.25 mg/mL). The measured MIC values for *Leccinum carpini *against bacteria were 1.25-5 mg/mL for the acetone and 2.5-10 mg/mL for the methanol extract. Both extracts of this mushroom inhibited the tested fungi in concentrations 5 mg/mL and 10 mg/mL.

The acetone and methanol extract of *Boletus aestivalis *and *Boletus edulis *had approximately equal antimicrobial activity. They inhibited the tested bacteria and fungi in concentrations 2.5 mg/mL, 5 mg/mL and 10 mg/mL.

The antimicrobial activities were compared to streptomycin (standard antibiotic) and ketoconazole (standard antimicotic). The results showed that streptomycin and ketoconazole had stronger activity than tested extracts as shown in Table 4. In a negative control, DMSO had no inhibitory effect on the tested organisms.

The tested mushrooms extracts have a strong antioxidant activity against various oxidative systems *in-vitro*. The intensity of antioxidant activity depended on the tested mushroom species and the solvent which was used for extraction. The differences in the antioxidant activity of various solvents may be result of different capabilities to extract bioactive substances (19). When antioxidative capacities of the extracts are compared to their phenolic constituents, it could be concluded that antioxidative nature of the extracts might depend on their phenolics. We found that the tested extracts exhibited the highest radical scavenging activity with the greatest amount of phenolic content. Numerous researches found a high correlations between antioxidative activities and phenolic content (8, 20, 21).

Antioxidant activity of some mushroom extracts were also studied by other researchers. In the literature there are numerous data for the antioxidant activity of *Boletus edulis *(22, 23, 24)**. **Similar results vere reported for some other mushroom. For example, Ramesh et al. (25) found strong antioxidant activity for methanole extracts from *Lycoperdon perlatum, Cantharellus cibarius, Clavaria vermiculris, Ramaria formosa, Marasmius oreades, Pleurotus pulmonarius. *Murcia *et al. *(26) find an antioxidant activity for the *Lepista nuda, Lentinus edodes, Agrocybe cylindracea, Cantharellus lutescens, *and *Hydnum repandum.*

Numerous mushrooms were screened for antimicrobial activity in search of the new antimicrobial agents (25, 27-29). It found that different species of mushrooms exhibit different antimicrobial activity. These differences in antimicrobial activity of different species of mushrooms are probably a consequence of the presence of different components with antimicrobial activity. 

In our experiments, the tested mushroom extracts and show a relatively strong antimicrobial activity. The intensity of the antimicrobial effect depended on the species of mushroom, its concentration and the tested organism. The examined mushroom in the same concentrations showed a stronger antibacterial than antifungal activity. These results could be expected due to the fact that numerous tests proved that bacteria are more sensitive to the antibiotic compared to fungi (30). The reason for different sensitivity between the fungi and bacteria can be found in different transparency of the cell wall (31). The cell wall of the gram-positive bacteria consists of peptidoglucans (mureins) and teichoic acids, while the cell wall of the gram-negative bacteria consists of lipo polysaccharides and lipopoliproteins, whereas, the cell wall of fungi consists of polysaccharides such as hitchin and glucan (32, 33).

## Conclusions

It conclusion, it can be stated that tested mushroom extracts have a strong antioxidant and antimicrobial activity *in-vitro*. Based on these results, mushrooms appear to be good and safe natural sources of antioxidants and could be of significance in making the food bad and in human therapy, animal and plant diseases. Further studies should be done on the isolation and characterization of new compounds from mushrooms, which are responsible for antioxidant and antimicrobial activity.
